# The Treatment of the Gouty State

**Published:** 1893-10

**Authors:** Frank Woodbury

**Affiliations:** Professor of Clinical Medicine in the Medico-Chirurgical College, formerly Attending Physician to the German Hospital of Philadelphia, etc.


### THE TREATMENT OF THE GOUTY STATE.

CLINICAL LECTURE DELIVERED BY FRANK WOODBURY, M. D.

Professor of Clinical Medicine in the Medico-Chirurgical College, formerly Attending Physician to the German Hospital of Philadelphia, etc.

Gouty patients should pay especial attention to the condition of the skin, and should avoid such exposure as would lead to chilling the surface and producing internal congestions. Great care should also be taken to keep the stomach in a normal condition, avoiding excess of eating or drinking, and all articles which are known to cause fermentation and flatulence. Occasional cholagogue purges are useful, especially with colchicum preparations, but too frequent purgation is debilitating, and should therefore be avoided. The kidney secretion must be watched and the appearance of a brick dust deposit should be regarded as a cautionary signal, to indicate either a deficiency of oxygen, hepatic failure, or errors of diet, or all combined. The occurrence of albuminuria should lead to the institution of proper measures to prevent the development of renal congestion, or degeneration (gouty kidney.)

Among the most important means of treating the gouty state is the persistent use of a good natural lithia water, which may be used at the table and at other times. As pointed out by Sir Henry Thompson, the artificial mineral waters or solutions of salts are less efficient therapeutically than waters from Nature's laboratory. Many mineral waters are offered to the public for lithaemic conditions, whose only therapeutic value resides in the water which they contain. Trousseau indeed cautions against an excess of water drinking by gouty subjects as being injurious and likely to intensify the symptoms. This is more likely to occur where the kidneys have been structurally altered by the disease, and therefore have their functional activity more or less reduced. Lithia on chemical grounds is considered the most useful of the alkali remedies, because it forms more soluble salts with uric acid than any other base. It is not clear however, why uric acid should combine with the lithia salts in the presence of sodium carbonates in the blood when as a rule the base which forms the insoluble salt is considered to have a stronger affinity for an acid than one which combines with it and remains in solution. I mention this because I am not willing to concede all the good results from such a water, for instance, as the well known Buffalo Lithia Water, to the presence of the lithia, any more than I would be to ascribe it entirely to the pure water which it contains. The fact is that experience has abundantly shown that the good effects of the natural lithia water are to be ascribed to the peculiar combination of salts, just as in drug prescribing we obtain special effects by combining our remedies. It is gratifying to witness the good effects of the daily use of four or five glasses of the Buffalo Lithia Water in relieving the usual symptoms of lithaemia, or even in removing the more severe condition of gravel or uric acid deposits. At the same time, I do not wish to be misunderstood. I think that at the present day there is danger of our taking too narrow a view of the pathology of gout, and to sum it up as simply uriaemia or lithaemia (lithiasis of Fothergill,) or an increased amount of uric acid in the system. This is an unfortunate error. Uric acid is not the cause of gout ; *it is the gout, on the contrary, which causes the excess of uric acid.* In other words, the gouty diathesis or condition is that in which there is a tendency to increased formation of uric acid, and also of oxalates, and it is accompanied by other well-marked pathological occurrences which complete the clinical picture. In several other disorders we have as great or even greater development of uric acid in the blood without the typical and classical tissue changes of podagra, and in gout the amount of uric acid is not the sole measure of the intensity of the gouty manifestations. The nervous phenomena of gout must not be overlooked, and the fact that heredity plays such an important part in its production, and its well recognized diathetic character, give much support to the view of the essentially nervous character of the disease.

Sir William Roberts has, in a recent lecture, shown that in gouty subjects the uric acid exists in the blood in the form of the soluble quadrurates, while in gouty patients it exists in the less soluble form of bi-urates ; the sodium bi-urate being particularly insoluble. Where this sodium bi-urate is in process of deposition in the form of tophi in the parenchyma of organs like the kidneys, in the articular surfaces of joints, and around them ; and in the pinnae of the ears, it is conceivable that a remedy like piperazine which exercises an extraordinary solvent effect upon the urates, might be useful for a rapid effect. But a rational treatment would be to shut off the supplies of soda and to diminish its total quantity in the blood. Acting upon this, Dr. Roberts has for years been in the habit of advising his gouty patients to restrict the use of salt with their meals, especially since table salt or sodium chloride in solution has the power to precipitate sodium bi-urate and increase the deposits in the joints. It is worth noting that butcher's meat contains considerable proportion of the chloride of sodium, while salt or pickled meat, or fish, is especially objectionable. Jonathan Hutchinson limits the amount of fresh fruits to be eaten by gouty patients, and especially of acid fruits, which are usually eaten with sugar. Some vegetables contain a decided portion of sodium salts, notably, white potatoes. While on the subject of diet I might say that recent authorities upon the treatment of irregular gout—and I refer especially to a recent lecture by Prof. DaCosta—limit the quantity of sugars and starches (carbonhydrates) in the food, chiefly on account of the tendency to the occurrence of acetous fermentation in the stomach in gouty patients. The former interdiction of nitrogen-containing foods is now removed, at least sufficiently to permit a varied dietary, one that is adapted to the digestive capacity, and to keeping up the forces of the economy. An excess of albumen, however, is to be carefully avoided so as not to throw too much work upon the kidneys.

Abstract from Helbring's Ph. Record, June '93 number:

Dr. F. W. Passmore and myself have conducted a series of examinations on Cocaine Preparations.

The accruing advantages obtained from systematic investigation of these bases are readily recognized.

There is hardly any alkaloid compound which is found in commerce in more varying degrees of purity than Cocaine, and none which is more important to obtain pure.

It gives me pleasure to place on record the fact that Cocaine preparations, and especially the Hydrochlorate of Cocaine, of C. F. Boehringer & Sochne, have been found free from all impurities; they answer all the tests of the Pharmacopoeias and yield definite unvarying physiological action without secondary effects.

It can only be by patient investigation, vigilantly pursued, that this Manufactory has attained the high standard of purity reached by the preparations we have reported upon.

H. Helbring, T. C. S., Analytical and Consulting Chemist, 63 Queen Victoria St., London, E. C.

---

LISTER'S RENEWED ALLEGIANCE TO CARBOLIC ACID.—It is not surprising to those who have kept pace with the progress of Antiseptics to hear Sir Joseph Lister, after twenty years' of investigation and experiment, declare his renewed allegiance to Carbolic Acid, as he did in a lecture January, 1893, at King's College Hospital in London (*Annals of Surgery*, June, 1893.)

Carbolic Acid is not only a more efficient surgical germicide than corrosive sublimate, but it is much more efficient in cleansing the skin. It has a powerful affinity for the epidermis, perpetrating deeply into its substances, and it mingles with fatty materials in any proportion. Corrosive sublimate on the other hand cannot penetrate in the slightest degree into anything greasy; whence those who use it require elaborate precautions in the way of cleansing the skin. All of this is unnecessary with carbolic lotion. Sir Joseph does not even use soap and water, trusting entirely to the Carbolic Acid.

There is a new product of Phenol and Boracic Acid, in which has been mitigated the pungency of Carbolic Acid, disguising its odor, and greatly supplemented its efficiency by its combination with Boracic Acid, which, although admittedly slower in its action, it nevertheless, unsurpassed as a true germicide.

